# Evaluation of the application potential of *Bdellovibrio sp.* YBD-1 isolated from Yak faeces

**DOI:** 10.1038/s41598-024-63418-9

**Published:** 2024-06-06

**Authors:** Yao Xi, Yangyang Pan, Mei Li, Qiaoying Zeng, Meng Wang

**Affiliations:** 1 College of Veterinary Medicine, Gansu Agricultural University, Lanzhou, China; 2 Technology and Research Center of Gansu Province for Embryonic Engineering of Bovine and Sheep & Goat, Lanzhou, Gansu China

**Keywords:** BALOs, *Bdellovibrio*, Yak, *Escherichia coli*, Biofilm, Bacteria, Biofilms, Pathogens

## Abstract

Studies on *Bdellovibrio* and like organisms (BALOs), obligate predatory bacteria, have highlighted the possibility of regulating bacteria and biofilms; however, yak-derived BALOs are yet to be reported. We aimed to characterize the BALOs isolated and identified from yak (*Bos grunniens*) feces and examine application potential. BALOs were isolated from healthy yak fecal samples, with *Escherichia coli* (ATCC 25922) as prey using the double-layer agar method, identified by transmission electron microscopy (TEM), and the specific 16S rDNA sequencing analysis. Sequencing of the 16S rDNA gene indicated that this isolate was 91% similar to the *Bdellovibrio sp*. NC01 reference strain and was named YBD-1. Proportion of YBD-1 lysed bacteria is 12/13. The YBD-1 showed best growth at 25–40°C, 0–0.25% (w/v) NaCl, and pH 6.5–7.5. YBD-1 significantly reduced the planktonic cells and biofilms of *E.coli* in co-culture compared to the *E.coli* group. Additionally, SEM analysis indicated that YBD-1 significantly reduced biofilm formation in *E. coli*. Furthermore, quantitative Real Time-polymerase chain reaction (qRT-PCR) showed that the expression of the virulence genes *fim* and *iroN* and the genes *pgaABC* involved in biofilm formation went down significantly. We concluded that YBD-1 may have the potential to control bacterial growth and biofilm-associated bacterial illnesses.

## Introduction

Antibiotics are extensively utilized to promote growth, illness prevention, and therapy in farmed animal industries^1–3^ Notably, the use of antibiotics often causes the emergence of drug resistance^1^, resulting in reduced or even extinguished efficacy of the therapeutic intervention against human and animal infections, causing a considerable challenge to public health, extensive food safety concerns, and environmental pollution^4–7^. Therefore, an urgent demand is for substitute antibiotic products to encourage animal development and avoid illnesses.

BALOs are Gram-stain negative bacteria that naturally invade and destroy Gram-stain negative and some Gram-stain positive pathogens^8–11^. They are widespread in natural and manufactured settings, including freshwater, seas, soils, plant rhizospheres, sewage, activated sludge, and human and animal intestines^12^. Since these predatory bacteria can eliminate a wide range of planktonic and biofilm bacteria without hurting higher eukaryotic species, they are being looked at as possible alternatives to antibiotics^13^ by significantly reducing their gene pools of antibiotic resistance^14^. Numerous in vitro toxicity studies and in vitro models have demonstrated that BALOs are not harmful^13,15,16^. Predatory bacteria are not only one of the most promising treatment options for multidrug-resistant diseases (MDR) infections^16^, but are also considered a significant mode of affecting bacterial community structure and microbial food webs^15^. Bacterial predation methods and characteristics have been thoroughly investigated in detail^17,18^. However, most current information is drawn from just a few model strains, resulting in a dearth of understanding of predatory bacterial species in the wild^19^.

Large ruminants known as yaks (Bos grunniens) are an essential source of life and income for highland farming communities in the Qinghai Tibet Plateau due to their strong ability to adapt to the region's high altitude, low oxygen concentration, low temperature, and lack of food^20,21^. Some researchers have even concluded that yaks have lived there for millions of years^22^. Yaks are not only used for farming and transportation but also provide residents with daily necessities, such as milk, meat, wool, leather, labor, and fuel, making them a vital mainstay industry for herders^20,23,24^. The ability of yaks to withstand harsh natural environments is related to their unique gastrointestinal micro-ecosystem^21,22^. Bacteria in the gastrointestinal system of yaks may have unique capacities due to changes in host species and living habitats^21,25^. Therefore, we were interested in isolating predatory bacterial BALOs from yaks.

The BALOs exhibit a lytic effect on many potential pathogens in nature, both planktonic cells and biofilms, by acting on specific virulence and biofilm-associated genes in the prey. Therefore, this study aimed to isolate and characterize BALOs from yak feces and to explore the prey range and lytic properties to assess the application potential of YBD-1.

## Materials and methods

### Collection, isolation, and purification of BALOs

#### Prey preparation

The bacterial strains employed in this investigation are enumerated in Table 1, and the strains without strain information are all laboratory isolates^26^. BALOs were isolated using *E. coli* ATCC 25,922 as prey. The prey bacterial (OD 2.0 at 600 nm) strains were cultivated for 1:100 in either Tryptic Soy Broth (TSB) or Luria–Bertani (LB) medium at 37°C with shaking at 220 rpm for 12–16 h (late exponential phase)^27,28^. After centrifuging the prey bacteria for 10 min at 4°C at 3000 g, the pellets were washed and resuspended in the diluted nutritional broth (DNB) (1:10 dilution of nutrient broth amended). After adjusting the prey cell suspensions to an optical density of around 2.0 at 600 nm (OD600) [~ 5 × 10^8^ CFU/ml], they were kept in storage at 4°C^28,29^.

**Table 1:** Assessment of prey range of *Bdellovibrio sp*. YBD-1. *Acinetobacter baumannii* was purchased from Biobw and the platform number is bio-53026.

Strain number	Species/strains	Strain information	Lysis by YBD-1
1	*Escherichia coli*	ATCC 25,922	Yes
2	*Salmonella typhimurium*	CMCC 50,115	Yes
3	*Streptococcus pyogenes*	ATCC 19,615	Yes
4	*Acinetobacter baumannii*		Yes
5	*Escherichia coli*	ATCC 700,728	Yes
6	*Staphylococcus aureus*	ATCC 29,213	Yes
7	*Staphylococcus aureus*	ATCC 25,923	Yes
8	*Staphylococcus aureus*	RN 4220	No
9	*Staphylococcus aureus*	TCH 1516	Yes
10	*Streptococcus agalactiae*	laboratory isolate (XN30-1)	Yes
11	*Staphylococcus haemolyticus*	laboratory isolate (XN47-2)	Yes
12	*Bacillus lichcheniformis*	laboratory isolate (FU81744-1)	Yes
13	*Bacillus subtilis*	laboratory isolate (3867–1)	Yes

## Sample collection, processing, and enrichment

Three samples of 20g each were collected from three-year-old adult healthy female yak (full of spirit, bright fur, free movement, no abnormality, and no signs of disease) fecal samples in Gannan of Gansu province, China, using sterile PE gloves, transferred to sterile 50 ml centrifuge tubes in dry ice, and sent immediately to the lab in 4h for further examination. One hundred milliliters of dilute nutrient broth (DNB) was added to 200 ml conical flask containing 20g samples, and to ensure that the organisms were distributed uniformly, the mixture was shaken with an orbital shaker set to 180 rpm for 1 h at 30°C^29^. Afterward, samples were incubated in an orbital shaker set to rotate at 180 rpm for an entire night at 30°C. The shaker was filled with 20 ml of washed *E. coli* prey cell suspension (prepared as mentioned above).

## Isolation and purification with *E.coli* ATCC 25,922

Samples were enriched overnight and filtered with sterile gauze to remove partial residual prey cells and impurity particles. They were sequentially centrifuged at 1500 g for 5 min at 4°C and 3000 g for 5 min at 4°C. The supernatant efficiently concentrated *Bdellovibrio*^28^. The 10 ml supernatant was mixed with 20 ml DNB. Then, a sterile 10 ml centrifuge tube served to combine 0.3 ml of the supernatant with 0.5 ml of the prey (*E.coli* ATCC 25,922), followed by incubation at room temperature for 30 min. Subsequently, 4 ml of 0.7% molten diluted nutrient agar was added to the potential BALOs mixture. BALOs were isolated and purified using double-layer agar plating. The plates were incubated at 30°C for 12 days while plaque formation on DNB agar was observed^29^. Plaques visible on DNB plates after 2–3 d and progressively expanding for several days were thought to be probable BALOs. One of the biggest plaques was lifted (the plaque was first dug with the end of the 1 ml pipet tip and then picked with the tip of the pipet tip) from the plate to a sterile 5 ml centrifuge tube to combine 0.6 ml of the DNB with 0.6 ml of the prey (Suspension of prey cells with DNB), followed by incubation at room temperature for 30 min. Finally, 1 ml of the mixed incubation was pipetted into a sterile 10 ml test tube with 4 ml of 0.7% molten diluted nutrient agar, and purification was carried out following the same procedure with double-layer agar plating. The plaques obtained were purified by individual plaque isolation techniques in at least three successive platings until lytic plaques on an agar plate were homogeneous in size^27,28,30,31^, and not other bacterial colonies appeared on the plate. We believe that purified BALOs have been obtained.

## Identification of the BALO

### Transmission *electron* microscopy

10 µl recently lysed attack-phase (~ 10^8^ PFU/ml) YBD-1 (the purified individual plaques were picked out and added to 100 ml of DNB containing 500 µl of *E. coli* ATCC 25,922 to incubate for 72 h at 30 °C, 180 rpm) suspension was placed on a Formvar carbon-coated 200-mesh copper microscope grid for 4 min at room temperature to determine the morphology of the BALOs. The excess liquid was removed using sterile filter paper. The samples were then counterstained with a 2% (w/v) phosphotungstic acid solution for 2 min before being viewed using a transmission electron microscope at a 100 kV accelerating voltage^28^ (Laboratory of Transmission Electron Microscopy, Lanzhou Veterinary Institute, Chinese Academy of Sciences).

## 16S rDNA

The BALOs' genomic DNA was extracted by centrifuging the co-culture twice at 3000 g for 5 min filtering the supernatant with a 0.45 µm bacterial filter to remove the remaining prey cells, then boiling the product for 10 min at 100°C and centrifuging at 12,000 g for 20 min. The supernatant obtained was used directly in the PCR reaction. PCR was used to amplify the *Bdellovibrio* 16S rDNA gene using primers specific to the *Bdellovibrio* 16S rDNA gene (63F primer: 5'-GAG GCC TAA CAC ATG CAA GTC-3'; 842R primer: 5'-CGW CAC TGA AGG GGT CAA-3')^32^. PCR amplification was performed by (Kodaq PCR MasterMix, Cat #G497) under the following conditions: predenaturation at 94°C for 10 min, 35 cycles of denaturation at 94 °C for 30s, annealing at 50°C for 30s, extension at 72°C for 48s, and final extension at 72°C for 10 s. The amplified products were analyzed in 1% agarose gel and identified with a UV transilluminator. The desired amplicon was extracted and purified using a Gel Purification Kit (TIANGEN, DP214-03) and sent for sequencing (Sangon Biotech, China, Shanghai).

## Phylogenetic analysis

To clarify the specific evolutionary relationship of the isolate with other BALOs, the gene sequence of one representative BALO isolate was acquired and deposited in GenBank with the accession number OR186335 and named *Bdellovibrio sp*. strain YBD-1 (OR186335). The Basic Local Alignment Search Tool (BLAST)^33^ application compared data to the National Center for Biotechnology Information (NCBI) database. The YBD-1 isolate's sequences were aligned with reference strains using ClustalW^34^. MEGA 7.0^35^ software was used to analyze the aligned sequences. The neighbor-joining method was used to build a phylogenetic tree. Bootstrap analysis with 1000 replications was used to determine the statistical significance levels of the inner nodes.

## Prey range determination

**Figure 1:**
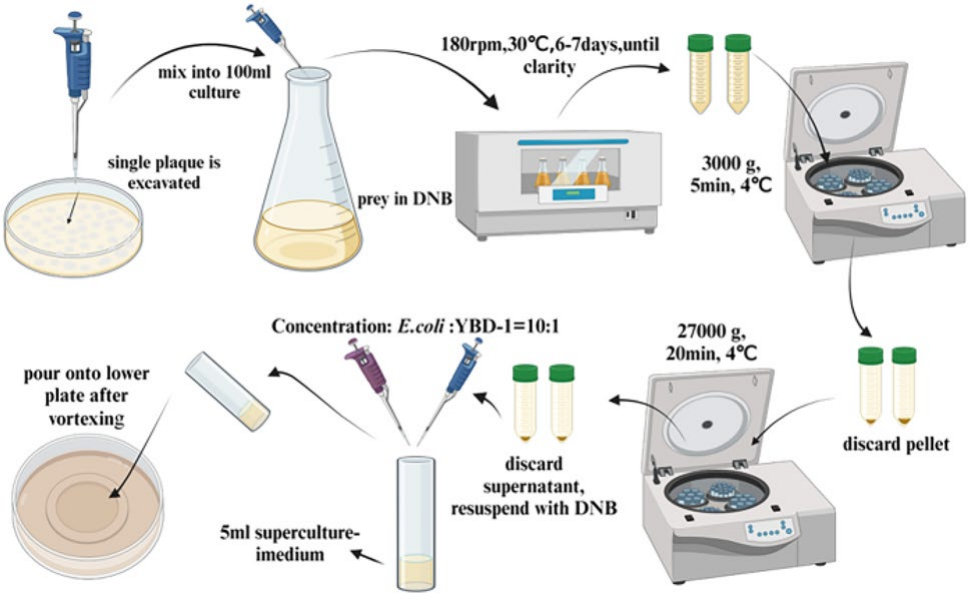
This is a flowchart for assessing of prey range of *Bdellovibrio sp*. YBD-1.

A single pure plaque was excavated and mixed into a 100 ml culture containing bacterial prey cells (culture method was the same as prey preparation section, and LB medium was used) that had been washed with DNB using a pipette tip to assess the lytic range of YBD-1. The culture was shaken at 180 rpm for 6–7 days at 30°C on an orbital shaker until liquid clarity (predator stock for 5 × 10^8^ and 5 × 10^9^ PFU/ml). Then, the culture was centrifuged for 5 min at 3000 g, 4℃, discarded the pellet again, centrifugeed the supernatant, and followed the previous method. A few milliliters of DNB medium were resuspended from the pellet to a final concentration of 1 × 10^6^ to 5 × 10^6^ cells/ml (PFU/ml of the predator by double plate counting)^36^. The prey (Table 2) -to-predator ratio was adjusted to 10:1, and the double-layer plating technique was used to determine the prey range, as previously described^26^. Specific steps as shown in Fig. 1.

**Table 2:** The effect of temperature on the growth and lytic activity of YBD-1 indicated by changes in the plaque diameter on the double-layer agar plating culture.

YBD-1	15℃,Plaque diameter (mm)	20℃,Plaque diameter (mm)	25℃,Plaque diameter (mm)	30℃,Plaque diameter (mm)	35℃,Plaque diameter (mm)	40℃,Plaque diameter (mm)	45℃,Plaque diameter (mm)	50℃,Plaque diameter (mm)
Day1	6.15 ± 0.25	6.29 ± 0.09	8.48 ± 0.24	9.16 ± 0.23	8.45 ± 0.32	7.77 ± 0.24	7.31 ± 0.33	6.29 ± 0.15
Day2	6.58 ± 0.59	7.693 ± 0.72	9.71 ± 0.06	13.64 ± 0.54	11.59 ± 0.44	9.52 ± 0.40	8.49 ± 0.48	7.21 ± 0.23
Day3	7.28 ± 0.32	8.52 ± 0.39	10.75 ± 0.43	17.47 ± 0.45	15.82 ± 0.66	10.35 ± 0.19	9.39 ± 0.14	7.61 ± 0.33
Day4	7.75 ± 0.38	9.73 ± 0.49	12.79 ± 0.60	21.51 ± 0.06	18.34 ± 0.45	12.52 ± 0.32	10.74 ± 0.71	8.07 ± 0.15
Day5	8.45 ± 0.29	10.51 ± 0.39	16.21 ± 0.06	27.34 ± 0.31	25.26 ± 0.11	14.52 ± 0.22	12.56 ± 0.19	8.36 ± 0.18

Values expressed as Mean ± SD.

## Lysis properties of YBD-1

### Temperature, salinity, and pH effects on YBD-1 predation

**Figure 2:**
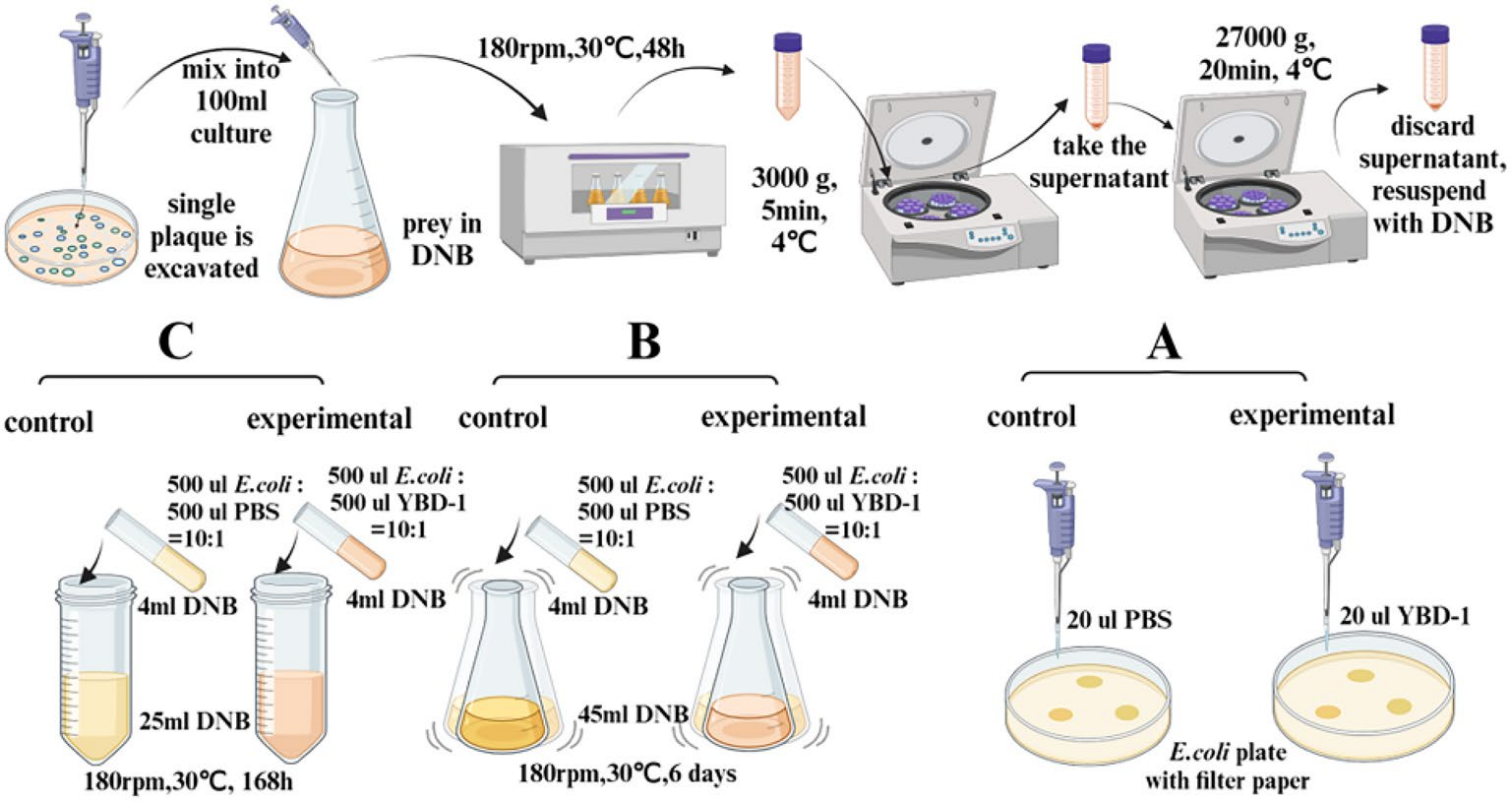
This is the flowchart. The effect of temperature (**A**, 15℃–50℃, with intervals of 5℃), pH (**B**, 5.0–9.0, with intervals of 0.5), and **.** salinity(**C**, 0.0, 0.25%, 0.5%, and 1.0%) on lytic activity of *Bdellovibrio sp*. YBD-1.

The temperature impact on YBD-1 was evaluated using the procedure described in section "Prey range determination" to get YBD-1. 20 µl of YBD-1 was dropped onto an *E.coli* plate with filter paper, and temperatures were set to 15°C-50°C, with intervals of 5°C, and the control experiments were set up with the same temperature gradient substituting YBD-1 for PBS. The pH impact to YBD-1 was achieved at 30°C in the same way as mentioned for the determination of lytic to incubated on a shaker (180 rpm for 48 h at 30°C) to gain predator. Five milliliters of *E. coli* suspensions (~ 10^9^ CFU/ml) contained 500 µl of predator in the attack phase (~ 10^8^ PFU/ml). The mixed cultures were incubated in 50 ml of DNB for 6 d at 180 rpm at a range of pH values (5.0, 5.5, 6.0, 6.5, 7.0, 7.5, 8.0, 8.5, and 9.0)^27,28^, and the control experiment was set up with the same pH gradient in the absence of predators. MOPS was used as the medium buffer to maintain the experimental pH. The salinity effect was detected at 30℃ with varying salinity (0.0, 0.25%, 0.5%, and 1.0%)( ignore the salt concentration of the medium itself ) in the same procedure mentioned with 30 ml mixed cultures for the pH effect. At the same time, the PFU/ml of YBD-1 was detected at 0 h and 168 h. Specific steps as shown in Fig. 2.

## Predation assays on planktonic cell cultures

As bacteria are mostly planktonic cells at the initial stage of infection, we measured the elimination of planktonic *E. coli* organisms by liquid culture with YBD-1, with slight modifications^37^. Briefly, 30 ml of DNB with 1 ml (1 × 10^8^ CFU/ml) of *E.coli* and 1 ml of recently lysed attack-phase YBD-1 (suspend YBD-1 with phosphate-buffered saline (PBS)) suspension (1 × 10^7^ PFU/ml) were incubated for 102 h at 30℃. A 30 ml DNB control culture containing *E.coli* at 1 × 10^8^ CFU/ml and 1 ml of PBS. Aliquots (1 ml) of the cultures were collected at 12 h intervals starting at 0 h. In total, 900 µl was used to measure absorbance values at OD600, and the viable cells of *E.coli* on LB plates were calculated using 100 L of dilution.

## Evaluation of YBD-1 impact on *E. coli* ATCC 25,922 biofilm

We evaluated the impact of YBD-1 on preformed biofilms and biofilm formation in *E. coli*. The effect of YBD-1 on preformed biofilms was determined, as described previously, with slight modifications^38,39^. Briefly, 200 µL of LB broth was added to a 96-well dish with an overnight culture of *E. coli* diluted 1:100 that was incubated at 30°C for 24, 48, and 72 h, and then washed using PBS to wipe out planktonic cells. Subsequently, 96-well dish was supplemented with 200 µl YBD-1 (1 × 10^8^ PFU/ml) or DNB for 24h. Biofilm-formation experiments were also performed. Briefly, the 96-well plates were inoculated with 200 µl of *E. coli* as prey to resuspend YBD-1 (2 × 10^7^ PFU/ml) as predator, and the plates were incubated for 24, 48, and 72 h. After incubation, the planktonic cell cultures and media components were removed using PBS. Then, the wells were dyed with 0.1% crystal violet (CV) for 20 min, and the unwashed dye was washed away with PBS by three washing steps. Finally, further determination was performed using the 30% acetic acid, and the OD600 was read as the absorbance of CV dye attached to the biofilm.

## Scanning electron microscopy (SEM) characterization of *E. coli* ATCC 25,922 biofilms

As described previously, a biofilm of *E, coli* was established on a 28-mm cell crawler in a 6-well microtiter plate^40,41^. Briefly, the experimental wells of the treated biofilm group contained 1 ml of an overnight culture of *E, col*i diluted 1:100 in LB medium with 1 ml YBD-1 (1 × 10^8^ PFU/ml). The control wells contained the same *E, coli* as above. The samples were fixed with 2.5% glutaraldehyde for 30 min at room temperature after 48 h of culture at 37°C. The samples were dehydrated gradient with ethanol (approximately 15 ml each), followed by drying at room temperature. Finally, the processed samples were sputtered with gold using an ion sputterer and viewed using an SEM at Wuhan Service Technology Co., Ltd. The voltage was adjusted to a value of 15 kilovolts (kV), and the samples were viewed with a magnification of 15,000 × .

## Quantification of virulence and biofilm formation gene expression with qRT-PCR

The impact of YBD-1 on the expression of virulence and biofilm-forming genes in *E. coli* has been assessed using quantitative reverse transcription PCR (qRT-PCR), following the previously established method^40^, with slight modifications. *E. coli* were cultured in LB medium with or without YBD-1 for 24 h at 37°C to isolate RNA. Before extracting RNA, we centrifuged for 5 min at 3000 g to get sediment, then executed *E. coli* cell counting experiments, the final adjustment of *E. coli* concentration. TRIzol reagent (Thermo Fisher Scientific 15,596,026) was used to extract total RNA from 1ml of culture. According to the manufacturer's instructions, the extracted RNA samples were reverse transcribed into cDNA using a cDNA synthesis kit ( TaKaRa RR047A) for qRT-PCR analysis. qRT-PCR was used to measure gene expression levels using a Recycle 96 system (Light Cycler 96) and the ChammQ SYBR qPCR Master Mix (Vazyme Q311-02), with the primers mentioned in Table 3. The 16S rRNA gene of *E.coli* was employed as an internal control to standardize the data^42^.

**Table 3:** Primer used for expression analysis of virulence genes and biofilm-forming genes in *E.coli* ATCC 25,922.

Function	Genes	Primer sequence(5,-3,)	References
Iron acquisition system	*IroN*	F: AAGTCAAAGCAGGGGTTGCCCG R: GACGCCGACATTAAGACGCAG	^43^
Adhesins	*fim*	F: GAGAAGAGGTTTGATTTAACTTATTG R: AGAGCCGCTGTAGAACTGAGG	^43^
	*pagA*	F: TCTTGCGGCGTATATTGGTAGGT R: CGACCCGACAATCACCAGTACG	^44^
Biofilm formation	*pgaB*	F: CGACGAAATGCGGCAATAACAC R: GCGGCGGCATATATTGTGGAAC	^44^
	*pgaC*	F: TCACCATCGGGATCAGCAAAT R: GCAGCAGAATACCGGGAAAGA	^44^

## Statistical analysis

MEGA 7 software was used to build the phylogenic tree, and Originpro 2019b and GraphPad Prism 8.0 were used to plot the diagram. The data was presented as mean ± standard deviation (SD) of at least three independent experiments. With SPSS 27.0, statistical analysis was carried out. One-way analysis of variance with the least significant difference (LSD) and multiple comparisons with Waller-Duncan treatment were carried out. It was deemed statistically significant when *P* < 0.05.

## Results

### Isolation and identification for *B. bacteriovorus*

**Figure 3:**
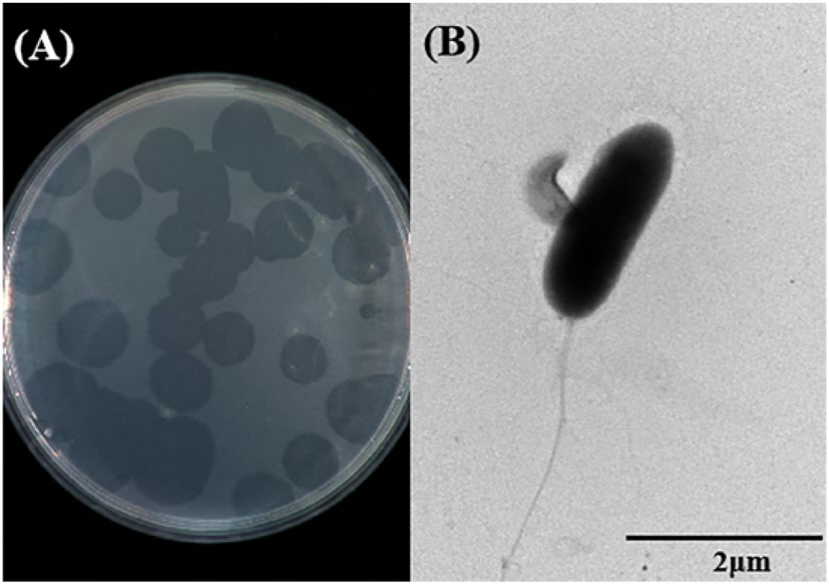
(**A**) Lytic plaques developed by YBD-1 on the lawn of *E. coli* prey cells. (**B**) Morphological identification of isolated YBD-1 in the attack phase using a transmission electron microscope(TEM). The scale bar represents 2.0μm.

**Figure 4:**
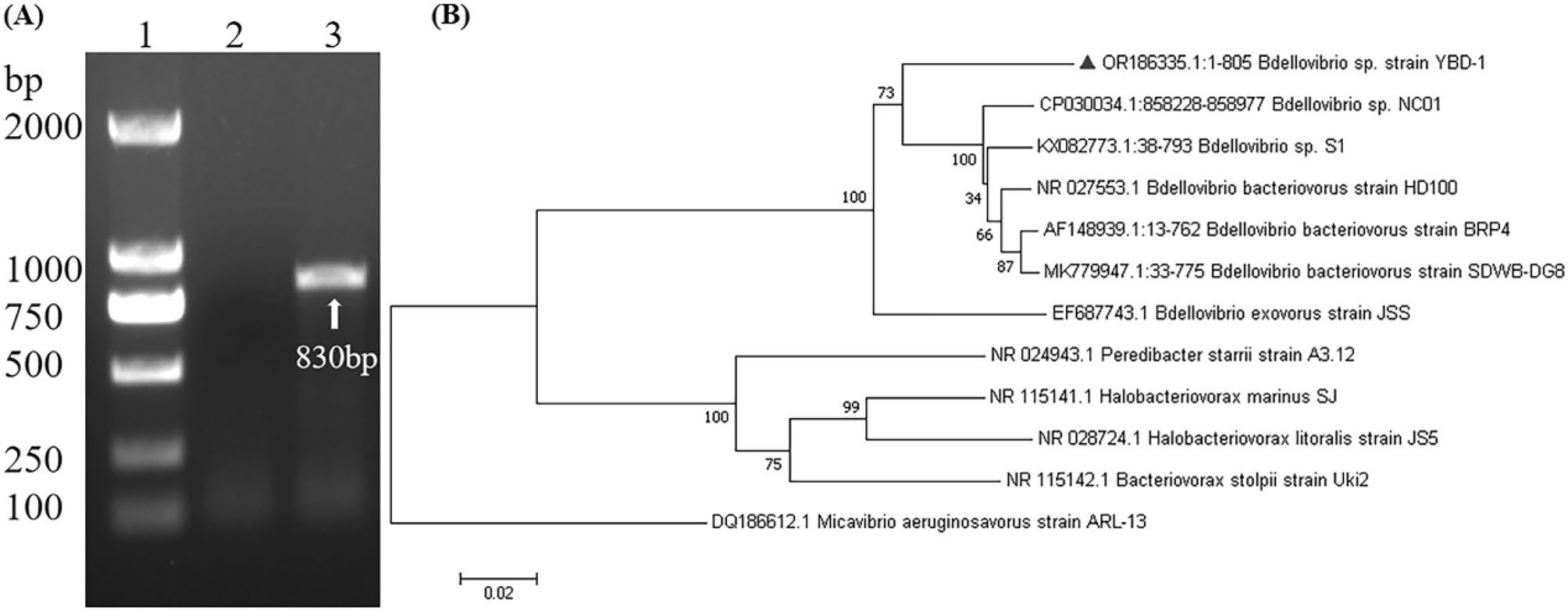
(**A**) Agarose gel electrophoresis of PCR assay using universal specific 16S rRNA gene primers (63F-842R), 1: DNA ladder marker(2 k bp), 2: *E.coli* negative control, 3: YBD-1 genomic DNA. (**B**) The phylogenetic tree is based on the specific 16S rRNA gene sequences of *Bdellovibrio*. The tree was constructed using the Neighbour Joining method and the p-distance model. Bootstrap confidence was calculated from 1000 replicates.

The YBD-1 was isolated from the Yak feces sample. Within 7 days, several lytic areas of *E. coli* prey were formed on the law of incubation at 30°C by the double agar plates technique (Supplementary Fig. 1). One of the larger plaques was chosen for further purification analysis in the same manner as for isolation. In purification experiments, we observed clear circular plaques of uniform size with uniform borders on the plates in Fig. 3A. TEM indicated that the isolated YBD-1 was a tiny comma cell (). The specific 16S rRNA gene was amplified using PCR with the primers (63F-842R) for further identification, and the amplified fragments (830 bp, Fig. 4A) were sequenced and revealed to be 91% similar in sequence to the *Bdellovibrio sp*. NC01 reference strain from GenBank^33^. A comparison of the phylogenetic tree analysis with the eleven BALO reference strains in GenBank revealed that the strain YBD-1 was near to distance as *Bdellovibrio sp* (Fig. 4B). The nucleotide sequence of 16S rDNA from the YBD-1 isolate has been deposited in the GenBank database with the following accession number: *Bdellovibrio sp*. strain YBD-1 (OR186335)^45^.

## Prey range determination

The prey range of YBD-1 determined based on plaque formation can be seen in Table 1, exhibiting that the *Bdellovibrio* strain YBD-1 was able to form plaques with 12 of the 13 (92%) bacteria tested, which suggests a wide prey range^36^.

## Effect of temperature, salinity, and pH on lysis properties

**Figure 5:**
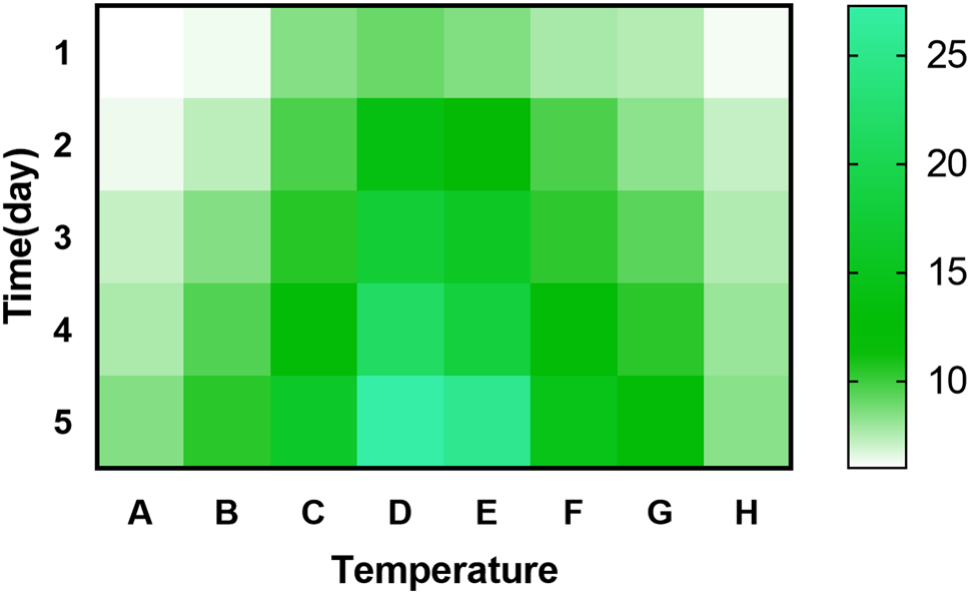
The effect of temperature on the lytic activity of YBD-1 indicated by changes in the plaque diameter(mm) on the double-layer agar plating culture. (A: 15℃; B: 20℃; C: 25℃; D: 30℃; E: 35℃; F: 40℃; G: 45℃; H: 50℃).

**Figure 6:**
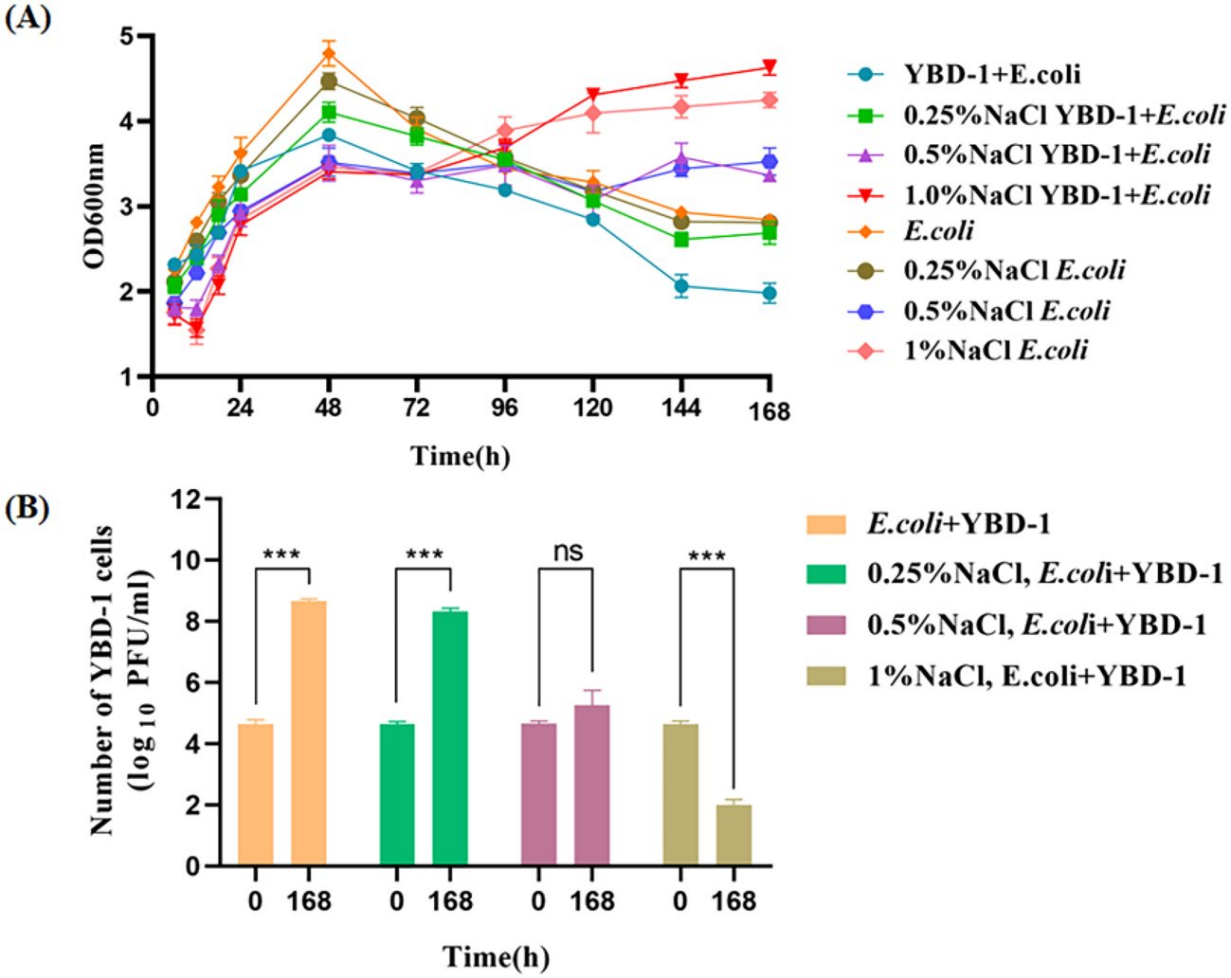
(**A**) The effect of salinity on the lytic activity of YBD-1 indicated by changes in OD600 in the broth co-cultures(30℃, pH 7.0 for 168 h). (**B**) Number of YBD-1 cells (log_10_PFU/ml) for initial starting time point 0 h, and final point 168 h. Salinity concentrations were tested at 0, 0.25, 0.5, and 1% NaCl, it is the same for Fig. 4A and B. All experiments were conducted three times independently. Statistical analysis was performed using two-way ANOVA. The amount of asterisks(*) obtained from SPSS is directly related to statistical significance(***: p < 0.001, ns: not significant).

**Figure 7:**
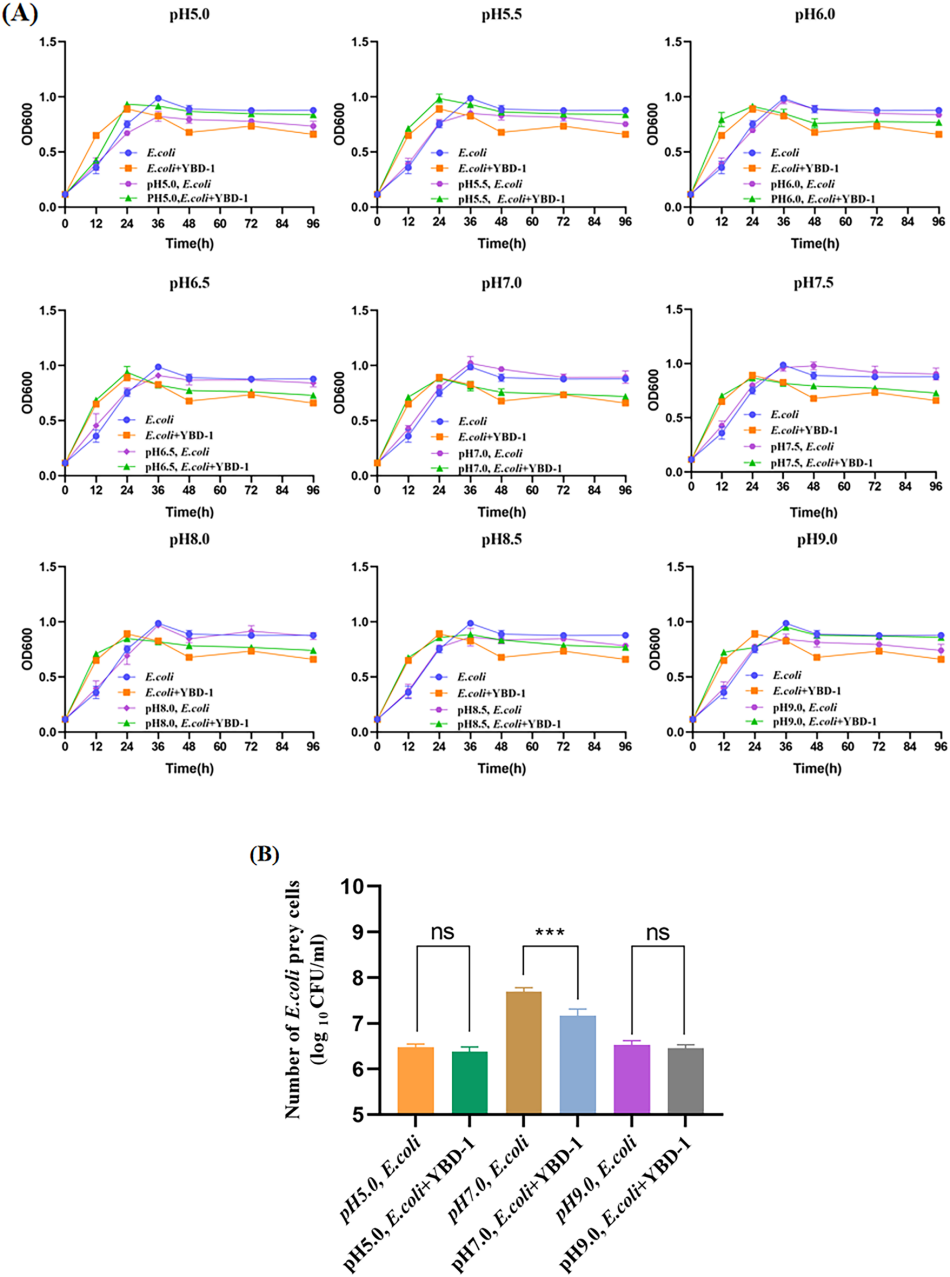
(**A**) The effect of pH on the and lytic activity of YBD-1 indicated by changes in OD600 in the broth co-cultures(30 ℃, pH 7.0 for 96 h). pH range was tested at 5.0, 5.5, 6.0, 6.5, 7.0, 7.5, 8.0, 8.5, and 9.0. (**B**) Number of *E.coli* prey cells(log_10_CFU/ml) with/ without YBD-1 for pH5.0,7.0,9.0 at 48 h. All experiments were conducted three times independently. Statistical analysis was performed using two-way ANOVA. The amount of asterisks(*) obtained from SPSS is directly related to statistical significance(***: p < 0.001).

Temperature (15°C–50°C), salinity (0.25–1%), and pH (5.5–9.0) were tested as typical environmental parameters on the predation activities of the isolated strains YBD-1. The method is to determine inhibition zone diameters with filter papers containing strain YBD-1 at 15°C, 20°C, 25°C, 30°C, 35°C, 40°C, 45°C, and 50°C. The largest diameter for the zone of inhibition was observed for strain YBD-1 at 25°C − 40°C and occurred within 5 d. At higher and lower temperatures, the circle of inhibition gradually decreased (Fig. 5, Table 2)^28^. Incubation experiments at the salinities ranging from 0%, and 0.25% salinity gradually decreased lytic ability; lytic ability is nearly aborted completely by 0.5% NaCl based on Fig. 6B, while at 1% NaCl, even YBD-1 viability based on PFU numbers (Fig. 6B) is decreased, indicating that lower salinity was optimal for YBD-1 (Fig. 6A, B). The strain YBD-1 exhibited its highest lytic activity at pH 6.5–7.5 within 96 h. There was no lytic effect at pH 5.5 and 9.0 (Fig. 7). We can see that *E.coli* & YBD-1 has always a higher OD600nm at 12h compared to *E.coli* alone.

## Predation assays on planktonic cell cultures

**Figure 8:**
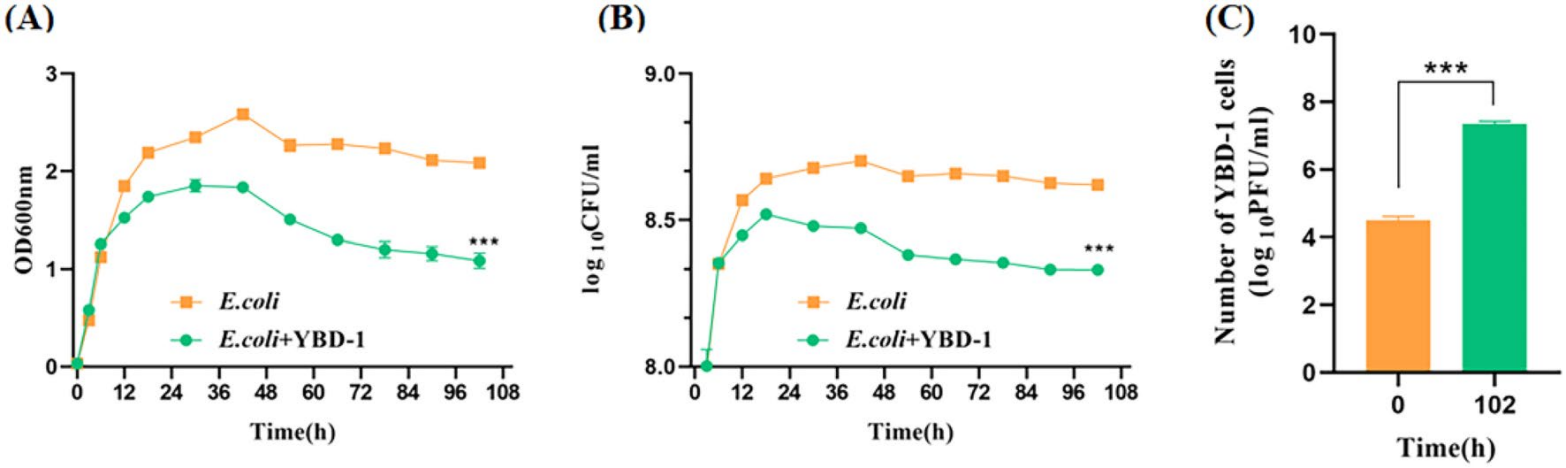
(**A**) OD600 of *E.coli* in co-culture with YBD-1; (**B**) log _10_ CFU/ml of *E.coli* in co-cultures with YBD-1. (**C**) log_10_FUP/ml of YBD-1 in co-cultures with *E.coli*. All experiments were conducted three times independently. Statistical analysis was performed using a paired t-test. The amount of asterisks(*) obtained from SPSS is directly related to statistical significance(***: p < 0.001).

Predation experiments on *E. coli* planktonic growth with and without YBD-1 indicated that YBD-1 exhibited predatory activity against *E. coli*. Predation viability was evaluated by determining the optical density and prey bacteria CFU/ml at 12 h intervals for 108 h. Figure 8 shows that after 12 h of co-culture with the predator YBD-1, there was a significant decrease in *E. coli* count compared to the control^37^.

## Impact on preformed biofilms and biofilm formation

**Figure 9:**
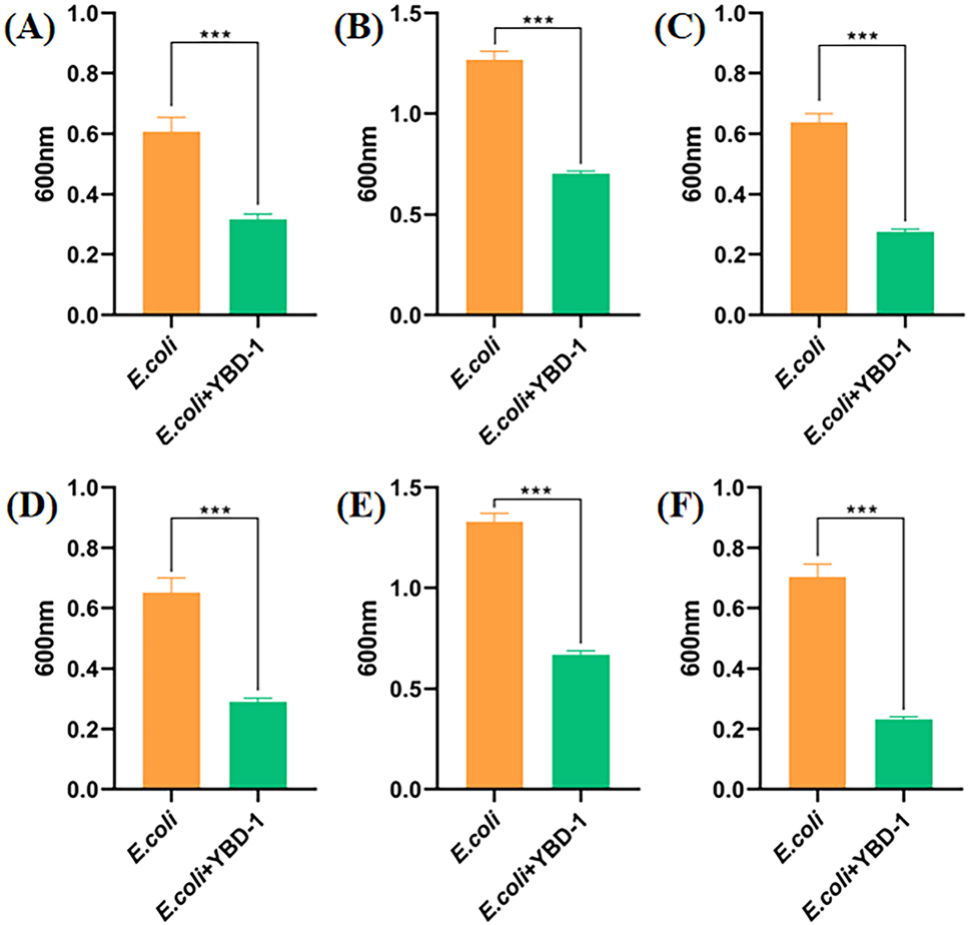
*Bdellovibrio sp*.YBD-1 predation assay on *E. coli* biofilm. (**A**) 24-h-preformed *E.coli* biofilm was treated with or without the predator(YBD-1) for 24 h; (**B**) 48-h-preformed *E.coli* biofilm was treated with or without the predator(YBD-1) for 48 h; (**C**) 72-h-preformed *E.coli* biofilm was treated with or without the predator(YBD-1) for 48 h. biofilm formation by *E.coli* after exposure to no or YBD-1 for 24 h (**D**), 48 h (**E**), or 72 h (**F**) All experiments were conducted three times independently. Statistical analysis was performed using a t-test. The amount of asterisks(*) obtained from SPSS is directly related to statistical significance(***: p < 0.001).

The impact of YBD-1 on preformed biofilms of *E. coli* was evaluated by dyeing with CVfor 24, 48, and 72 h biofilms incubated with a suspension of YBD-1 for 24 h. A significant reduction in biofilm biomass was observed compared to untreated control. Similarly, YBD-1 prevented the development of *E. coli* biofilms when co-cultured with predators and prey for 24, 48, and 72h, and compared to the control group, biofilm formation was significantly decreased (Fig. 9).

## Scanning *electron* microscopy (SEM)

**Figure 10:**
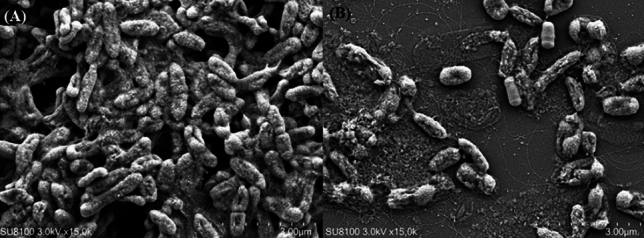
SEM image. (**A**) *E.coli* biofilm formation untreated with YBD-1 for 48-h; (**B**) *E.coli* after 48-h exposure to YBD-1.

To determine if YBD-1 affected the structure of the biofilms, we performed SEM investigations. The results showed that *E. coli* treated without YBD-1 form biofilms than *E. coli* treated with YBD-1 (Fig. 10).

## qRT-PCR quantification of virulence and biofilm formation gene expression

**Figure 11:**
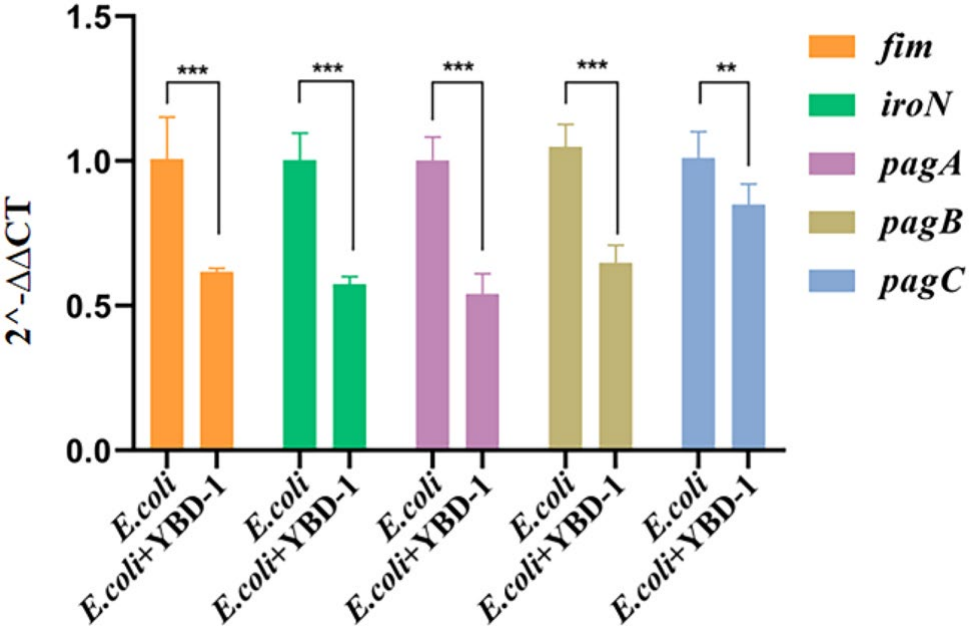
Expression ratio of *E. coli* virulence and biofilm formation genes in the presence or absence of YBD-1. All experiments were conducted three times independently. Statistical analysis was performed using two-way ANOVA. The amount of asterisks(*) obtained from SPSS is directly related to statistical significance(***: *p* < 0.001; **: *p* > 0.001). The 16S rRNA gene of *E.coli* was used as an internal control to normalize the data.

It has been proven that virulence factors contribute to *E. coli* pathogenicity^46^, and the first step for BALOs to lyse *E. coli* is through the action of the bacterial outer membrane^15^. The expression of virulence and biofilm-forming genes was studied (Table 3) in *E. coli* incubated with YBD-1 using qRT-PCR. The findings demonstrated a significant reduction in the expression levels of virulence and biofilm-forming genes in cells from cultures treated with YBD-1 compared to those in untreated cells (Fig. 11).

## Discussion

The emergence and use of antibiotics have provided enormous welfare for humans; however, their abuse also pollutes the human environment and causes significant health hazards. That is to say, the use of antibiotics does inhibit the occurrence of many diseases, but it has developed resistance to many pathogens. Therefore, researchers are interested in exploring alternative antibiotics, and predatory bacteria have become a focus of research.

Yaks, a rare species endemic to plateau regions, can adapt to harsh natural environments. Although predatory bacteria or BALOs have been studied in other animals^13,47^, they have not been reported in yak species. Therefore, this study aimed to isolate and characterize BALOs from yak feces and evaluate their potential application. We isolated a bacterial strain from yak feces that was consistent with the results of other studies and was also a new species. To assess the application potential of YBD-1, we conducted a few studies on lysis range, characteristics, biofilms, virulence, and biofilm formation gene expression.

Our procedure for successfully isolating and purifying YBD-1 is rather difficult, and various attempts were made in the early stages. In the first attempt the enrichment cultures passed subsequently through a 0.45 µm Millex filter^37,44^; The second try included centrifugation at different speeds, through 1.2 µm or 0.8 µm and 0.45 µm^30,31^; Finally, a protocol^27,48^ was performed involving centrifugation, filtration, and re-centrifugation. Transparent plaques were not observed on double-layer agar plates. After the enrichment culture, a sterile gauze was used to filter out large particles and centrifuged at 1500 g and 3000 g for 5 min. Eventually, we observed transparent plaques on the double-layer plate after approximately 5 d, which gradually increased in plaque size with the increase in days and finally formed transparent plaques with more regular borders (Fig. 3A). We suspect that other centrifugation and filtration methods may have removed the other prey while removing growth-dependent BALOs, leaving a minimal number of progeny BALOs. The viability of BALOs gradually disappeared during the purification process, resulting in the absence of BALO plaques. Similarly, we did not obtain transparent plaques when we selected single plaques for subculturing. We observed transparent plaques when we reduced the proportion of prey bacteria, and we hypothesized that the most important reason could be that the ratio of BALOs to the host reached a better ratio, as some earlier studies have shown that different ratios of prey can lead to different lysing affections^48,49^.

The strain identity was confirmed by electron microscopy (Fig. 3)and the *Bdellovibrio*-specific primers to amplify *Bdellovibrio*-specific 16S rDNA^32^. Evolutionary tree construction revealed that the strain YBD-1 was near to distance as *Bdellovibrio sp*, but is a new genus because its similarity is only 91% to the *Bdellovibrio sp*. NC01 (Fig. 4). The strain YBD-1 is 90.9% to Bdellovibrio bacteriovorus strain SDWB-DG8. Electron microscopy results showed that the isolated YBD-1 was comma-shaped.

The ability of *Bdellovibrio sp*. strain YBD-1 to work as a live antibiotic against infections is demonstrated by its bacteriolytic spectrum. We selected four Gram-stain negative and nine Gram-positive strains (Table 1) to determine the ability to lyse a wide range of pathogenic bacteria. *S. aureus* RN 4220 was absent from lysing plaques among the tested bacteria (Table 1, Supplementary Fig. 2). The predator must first approach and recognize suitable prey, then breach the outer membrane and colonize the prey’s periplasm. Once the breaching has occurred, prey and predator form a characteristic round-shaped structure called bdelloplast^47^. *S.aureus* RN 4220 has a mutation in the gene *sau1 hsdR*, which can cause RN 4220 to become a gene defect type of the restriction-modification system. Therefore, it is likely that the genetic defect of RN 4220 causes *Bdellovibrio* to fail to recognize prey or that the site on the outer membrane of RN 4220 that contacts *Bdellovibrio* fimbriae cannot be activated. Based on the size, transparency, and edge regularity of the lysis plaques, we noticed that the ability of YBD-1 to lyse Gram-stain negative bacteria was more potent than that of Gram-stain positive bacteria, and the lysis of *E. coli* ATCC 25,922 was the most effective. Therefore, subsequent experiments were conducted using *E. coli* ATCC 25,922*.* For strongly pathogenic bacteria, *Streptococcus pyogenes* ATCC 19,615, *Acinetobacter baumannii*, *Escherichia coli* ATCC 700,728*, and Staphylococcus aureus* TCH 1516 also have different lysis effects (Supplementary Fig. 2), which seem to have a more promising discovery for application, and we will focus on these critical pathogenic bacteria in future studies. Furthermore, researchers have concluded that BALOs are exclusive predators of Gram-negative bacteria^50–52^. However, in recent years, studies have rarely found that Gram-stain positive bacteria can also be lysed by BALOs^11,53^. There is some, but limited evidence that some Gram-stain positive organisms might be attached/killed. Therefore, YBD-1 lyses the tasted Gram-stain negative and partial-stain positive bacteria, according to our initial double-layer agar plating results. Our future research will mostly focus on studying Gram-stain positive species as prey. There have also been no reports of BALOs invading mammalian cells^54,55^. The proportion of YBD-1 lysed bacteria is 12/13, and the broad lysis spectrum supports the heterogeneity of BALOs from different sources. BALOs was isolated from different sources, which is the most trustworthy data confirming its safety and potential probiotic activity in animals and humans. Therefore, YBD-1 may be able to control a variety of bacterial infections.

The effects of temperature, salinity, and pH on YBD-1 predation efficiency were investigated. The observed temperature range of 25°C–45°C (Fig. 5, Table 2) and pH range of 6.0–8.5 are consistent with the results of earlier research^28,31^, but the ranges have expanded, which may be attributed to the different settings of the gradient for the determination of the temperature and pH as well as the difference 0–0.5% (Fig. 6A, B), in contrast to the optimal salinity of 2% reported in Kongrueng et al.^36^. However, the source of their samples was an aqueous environment, and it is reasonable that the salinity was also appropriately raised. The range of salinity detected by Li et al.^29^ was 1–4.5%, which was also shown to be isolated from a marine brine system.

Considering the final application pathway of YBD-1, we examined the lytic effect on planktonic *E. coli* in liquid culture. Compared to the control group, YBD-1 had a significant lytic effect on planktonic *E. coli* (Fig. 8). Biofilms increase bacterial tolerance to antibiotics, the environment, and host immune system attacks and provide a protective environment for pathogenic bacteria^47^; BALOs have been shown to act on biofilms^40^. Biofilm-forming bacteria and fungi have been estimated to cause 80% of all infections^44^. Therefore, we first proposed using BALOs to lyse *E. coli* biofilms. *E. coli* exhibited a decrease in biofilm biomass contrary to the control group. We determined the effectiveness of YBD-1 in disrupting preformed biofilms and biofilm formation using a 96-well plate, and YBD-1 showed a significant lytic effect on each treatment group (Fig. 9). In addition, to better visualize the biofilm pattern, we observed the predation behavior of YBD-1 using SEM (Fig. 8), and the results support and extend the results of earlier studies^38,40–42,44–47^.

It has been demonstrated that the pathogenicity of *E. coli* isolates is partially caused by virulence factors. *IroN* gene is a necessary component encoding iron acquisition systems for the survival of bacteria. During an infection, bacteria have evolved several methods for acquiring iron, including an iron acquisition system^43,44^. The type 1 fimbria necessary for bacterial adherence and bladder epithelial cell invasion is encoded by the *fim* gene cluster^43,56^. The pgaABC locus is necessary for biofilm formation in *E. coli*. We found a significant decrease in the expression of *pgaA*, *pgaB*, and *pgaC* after YBD-1 exposure in *E. coli* (Fig. 11)^44,46^. We again evaluated that BALO-related isolates inhibited the viability of *E. coli* when treated with YBD-1^40^. Therefore, we hypothesized that YBD-1 kills *E. coli* by inhibiting virulence and biofilm formation genes.

## Conclusion

The YBD-1 was isolated from yak feces and identified by TEM, PCR, and the 16Sr DNA gene sequencing analysis in this study, indicating the isolate as *Bdellovibrio sp*. YBD-1. Several experiments were conducted to investigate the application potential of the isolate by determining the range of prey, lysis characteristics of YBD-1, planktonic cells, and biofilm lysis, and identifying prey bacteria's virulence genes and biofilm formation-related genes. All results indicate that isolate YBD-1 has the potential to control bacterial growth and biofilm-associated bacterial infections. This work delivers in-depth insights into the differences in predation characteristics among wild-type environmental predation bacterial strains, which provides more possibilities for the development of alternative antibiotic products.

### Supplementary Information

Supplementary Figures.

## Data Availability

The nucleotide sequence of 16S rDNA from the YBD-1 isolate has been deposited in the GenBank database with the following accession number: *Bdellovibrio sp*. strain YBD-1 (OR186335). The datasets presented in the study are available in the article/Supplementary Material. Further questions should be forwarded to the corresponding authors.
