# Supplementary material for: Evaluation of the application potential of Bdellovibrio sp. YBD-1 isolated from Yak faeces
Source: Sci Rep. 2024 Jun 6;14:13010. doi: 10.1038/s41598-024-63418-9 (PMC11156984; doi:10.1038/s41598-024-63418-9)
Supplement: Supplementary file 1 — Supplementary Figures. [file 41598_2024_63418_MOESM1_ESM.docx]

**
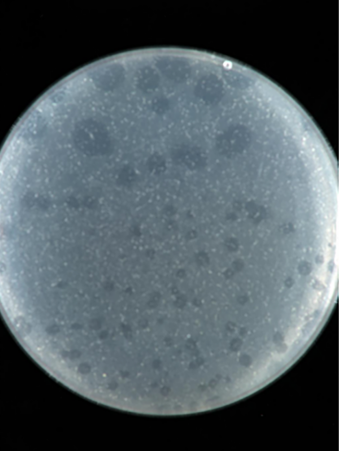
**

**Supplementary Figure 1.** YBD-1 Lysis plaques formed by the first isolation on the lawn of *Escherichia coli* ATCC25922 prey cells. The meaning of the first isolation was the first isolation of YBD-1 unpurified from treated yak faeces with *E.coli.*


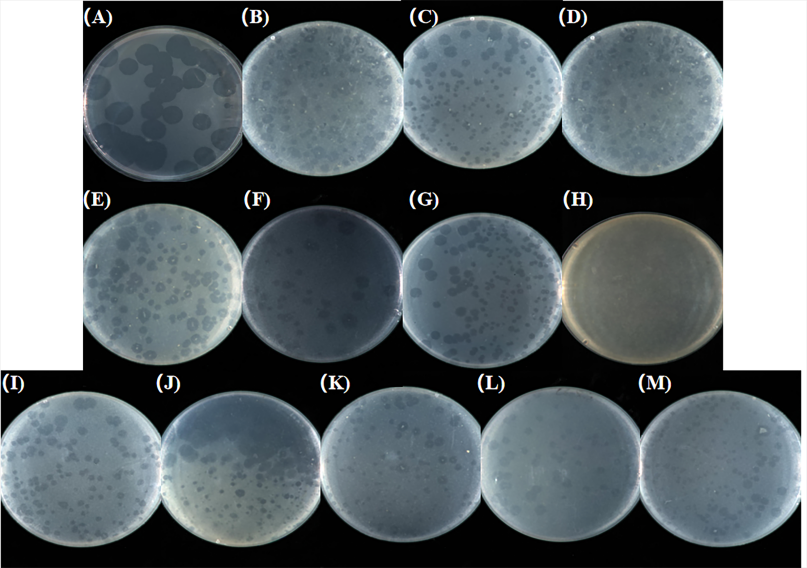


**Supplementary Figure 2.** The potential prey of YBD-1 and whether YBD-1 is sensitive to potential prey are listed in the table. (A): *Escherichia coli* ATCC25922; (B): *Salmonella typhimurium* CMCC50115; (C): *Streptococcus pyogenes* ATCC19615; (D): *Acinetobacter baumannii;* (E): *Escherichia coli* O157:H7; (F): *Staphylococcus aureus* ATCC25913; (G): *Staphylococcus aureus* ATCC25923; (H): *Staphylococcus aureus* RN4220; (I): *Staphylococcus aureus* USA300; (J): *Streptococcus agalactiae* laboratory isolate; (K): *Staphylococcus haemolyticus* laboratory isolate; (L): *Bacillus lichcheniformis* laboratory isolate; (M): *Bacillus subtilis* laboratory isolate.


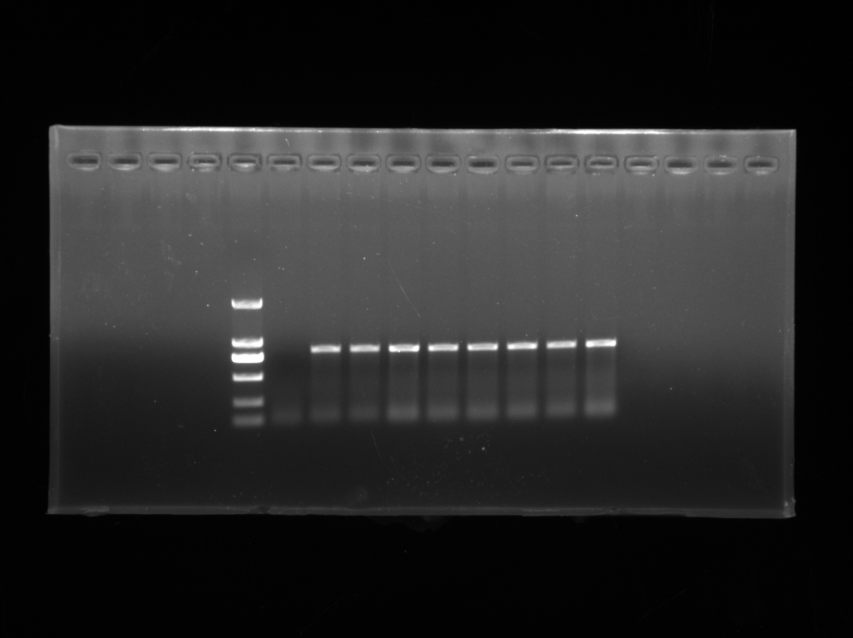


1 2 3 4 5 6 7 8 9 10

**Supplementary Figure 3.** Figure 4(A) Agarose gel electrophoresis of PCR assay using universal specific 16S rRNA gene primers (63F-842R), 1: DNA ladder marker(2k bp), 2: *E.coli* negative control, 3: YBD-1 genomic DNA. 4-10: Repeated amplification.
